# Polyvinyl Alcohol Composite Films Containing Flame-Retardant DOPO-VTES and α-ZrP

**DOI:** 10.3390/polym17081011

**Published:** 2025-04-09

**Authors:** Jiayou Xu, Minyi Luo, Riyan Lin, Shu Lv

**Affiliations:** School of Chemistry & Chemical Engineering, Guangzhou University, Guangzhou 510006, China; yangtaopeng516@163.com (M.L.); 18365563729@163.com (R.L.); thankxgod@163.com (S.L.)

**Keywords:** PVA, α-ZrP, DOPO, flame retardant, cross-linking

## Abstract

Polyvinyl alcohol (PVA) is used in various fields; however, its highly flammable property greatly limits its application. In order to improve the flame-retardant properties of PVA, one method is by adding flame retardants directly, while another method is through grafting, cross-linking and hydrogen bonding. A flame retardant, 9, 10-dihydro-9, 10-oxa-10-phosphaphenanthrene-10-oxide (DOPO)-vinyltrimethoxysilane (VTES), was synthesized through the addition reaction of a P–H bond on the DOPO and unsaturated carbon–carbon double bonds on the VTES. Then, the DOPO-VTES and zirconium phosphate (α-ZrP) were blended with PVA to cast a film, in which DOPO-VTES was grafted onto the PVA by cross-linking the hydroxyl group in the molecular structure of DOPO-VTES with the hydroxyl group in PVA; α-ZrP was used as a cooperative agent of DOPO-VTES. The cone calorimetry test (CCT) showed a significant reduction in both the heat release rate (HRR) and total heat release rate (THR) for the flame-retardant PVA films compared to pure PVA. Additionally, thermogravimetric analysis (TGA) revealed a higher residual char content in the flame-retardant PVA films than in pure PVA. These findings suggested that the combination of DOPO-VTES and α-ZrP could improve the flame retardancy of PVA. The cooperative flame-retardant mode of action at play was possibly that DOPO in the DOPO-VTES acted as a mainly gas-phase flame retardant, which yielded a PO radical; VTES in the DOPO-VTES produced silicon dioxide (SiO_2_), which acted as a thermal insulator; and α-ZrP catalyzed the carbonization of the PVA. By combining DOPO-VTES with α-ZrP, a continuous dense carbon layer was formed, which effectively inhibited oxygen and heat exchange, resulting in a flame-retardant effect. It is expected that flame-retardant films for PVA have a broad development prospect and potential in the fields of packaging materials, electronic appliances, and lithium-ion battery separators.

## 1. Introduction

Polyvinyl alcohol (PVA) is a water-soluble polymer with excellent film-forming performance, non-toxicity, biocompatibility and biodegradability. It is widely used in various fields such as textiles, construction, coating, packaging and electronic wearables [1]. PVA is extremely flammable and has an ultimate oxygen index of only 19%, owing to its structure, consisting of carbon, hydrogen and oxygen atoms [2].

The inherent flammability of PVA poses a high fire risk, so a lot of PVA products, including packaging materials, electrical and electronic appliances, and lithium-ion battery separators, must be flame-retardant. Therefore, improving the flame retardancy of PVA products is of increasing interest to researchers in the field of fire science. In order to improve the flame retardancy of PVA, researchers have used two kinds of flame-retardant methods. One method employs halogen-free flame retardants, including chemicals containing phosphorus (P)-nitrogen (N) [3], silicon (Si) and boron (B) [4]. Another approach enhances the flame retardancy of PVA by utilizing the interaction between PVA and flame retardants through grafting, cross-linking [5], chemical reaction and hydrogen bonding. For additive flame retardants, there are problems such as the compatibility between components and dispersion of additives in the PVA. This is because of the abundance of –OH groups in PVA, which can react with other active groups, such as –OH, –COOH and –CHO, used to improve its thermal stability, flammability and other properties. Wang [6] prepared PVA/guanidine phosphate (GP) films with high transparency based on the interaction of hydrogen bonds between PVA and GP. The PVA/GP flame-retardant membrane with high transparency had a 47% increase in tensile strength and its flame retardancy achieved a UL94 V-0 rating when the content of GP was 15 wt%. It was shown that the interaction between GP and PVA effectively promoted the formation of continuous and stable char in the condensed phase.

The widely used flame retardant DOPO exhibits mainly gas-phase activity, yielding a PO radical [7,8,9]. Some kinds of DOPO-modified alkoxysilanes have been developed by an addition reaction between the P–H bond of DOPO and the epoxy or olefinic groups of siloxanes [10]. Jelena Vasiljevic synthesized a novel flame retardant (DOPO-VTES) containing phosphorus-silicon and applied it to cotton fabrics at different concentrations using a sol-gel process. These flame retardation mechanisms can be ascribed to the combined activity of DOPO acting in both gas and condensed phases and silicon acting in the condensed phase [11].

Layered nanofillers, such as montmorillonite, have gained attention in recent years for improving the flame-retardant properties of polymers [12]. Alpha-zirconium phosphate (α-Zr(HPO4)_2_·H_2_O, α-ZrP) is a solid acid catalyst. α-ZrP nanosheets can enhance the carbonization degree of char residues and hinder toxic gas and heat transfer due to their well-organized lamellar structures. Consequently, they are often applied as functional additives in polymer matrices to improve flame retardancy [13]. Yuan Hu et al. used α-ZrP as a synergistic agent with phosphorus-nitrogen flame retardant ammonium polyphosphate (APP) to fire retardant polypropylene (PP) [14].

Therefore, in our work, α-ZrP was used as a cooperative agent of DOPO-VTES to create a novel flame-retardant system, with the aim of further improving the flame retardancy of DOPO-VTES for PVA film [15]. A phosphorus-silicon flame retardant (DOPO-VTES) was synthesized through an addition reaction between DOPO and VTES. Then, the DOPO-VTES was grafted onto the PVA structure through the cross-linking of the hydroxyl group in the molecular structure of DOPO-VTES with the hydroxyl group in PVA. The flame-retardant properties and flame-retardant mode of action of the PVA/DOPO-VTES/α-ZrP films were studied using the vertical burning test, the cone calorimetry test, SEM, XPS and Raman spectroscopy. Compared with additive flame-retardant PVA film [16], PVA/DOPO-VTES/α-ZrP films are expected to find a wide range of applications, especially in food packaging, electrical and electronic appliances, and lithium-ion battery separators.

## 2. Experimental

### 2.1. Materials

DOPO (white powder, purity ≥ 99.0%) was purchased from Shanghai Energy Chemical Co., Ltd. (Shanghai, China). PVA-1750, Analytical Reagent, with a polymerization degree of 1750, was purchased from Kelong Chemical Reagent Corporation (Chengdu, China). An amount of 99% of α-ZrP content was purchased from Xiya Reagent Co., Ltd. (Chengdu, China). An amount of 99% of vinyltrimethoxysilane (VTES) content was purchased from Shandong Sunway New Material Co., Ltd. (Zibo, China), and azobisisobutyronitrile (AIBN), the chemical reagent, was purchased from Sigma Aldrich (Shanghai, China). All the chemicals were used as received, without further purification, unless otherwise stated.

### 2.2. Synthesis of DOPO-VTES

**Scheme 1:**
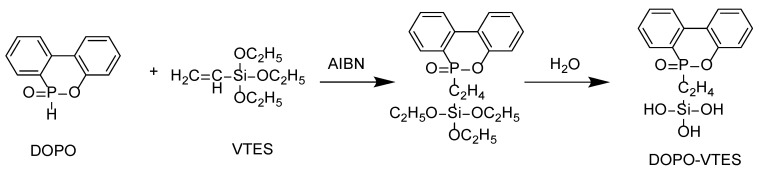
The synthetic route of DOPO-VTES.

Phosphorus-containing silane (DOPO-VTES) was synthesized through the addition reaction between DOPO and VTES, as described [17]. The typically, amounts of 21.6 g (0.1 mol) of DOPO and 19.0 g (0.1 mol) of VTES were mixed together in 50 mL of ethanol to obtain a transparent mixture. In the presence of radical initiator AIBN, at 75 °C for 6 h, a P–H bond on DOPO and unsaturated carbon–carbon double bonds on VTES underwent an addition reaction. Afterward, 10 g of distilled water was added, and the mixture hydrolyzed for 1 h, from which a light yellow viscous liquid was obtained. The synthesis of DOPO–VTES is presented in Scheme 1.

### 2.3. Preparation of the PVA Composite Films

1. An 8 wt% PVA solution was obtained by adding 8 g of polyvinyl alcohol powder and 92 g of distilled water to a round-bottom three-mouth flask. It was first swelled for 1 h, then stirred at 95 °C for 2 h, and finally cooled at room temperature.
2. According to Table 1, the PVA solution was mixed with the synthesized phosphorus-silica flame retardant (DOPO-VTES) and the different mass fractions of α-ZrP. The mixture was poured into surface dishes and cross-linked at room temperature to form a PVA composite film.

### 2.4. Characterization

Fourier-transform infrared spectroscopy (FTIR) was performed using a Nicolet 6700 spectrometer (Thermo Scientific, Waltham, MA, USA) in the frequency range of 500 to 4000 cm^−1^ with a resolution of 4 cm^−1^ to detect the functional groups on DOPO, VTES and DOPO-VTES.

^1^H NMR and ^29^Si-NMR spectra were obtained on a Varian Unity INOVA 300 MHz Upgrade spectrometer at room temperature with CDCl_3_ as the solvent and tetramethylsilane (TMS) as the internal standard.

Differential scanning calorimetry (DSC) measurements were interfaced with a TA 2010 (TA instruments, New Castle, DE, USA) under a N_2_ atmosphere with a heating rate of 10 °C/min at temperatures ranging from 30 to 300 °C. Thermograms were recorded during the first heating cycle and then cooled to 20 °C.

The morphology of the films was analyzed by a scanning electron microscope (JSM-6380LV, JOEL, Tokyo, Japan). The surface treatment involved gold coating before recording the samples. Surface elemental compositions were analyzed using energy-dispersive spectrometry (EDS) (INCA, Oxford Instrument, Abingdon, UK).

Thermogravimetric analysis (TGA) was performed on a TA Instrument (TGA Q50) from 50 °C to 700 °C at a heating rate of 10 °C/min. The samples (about 5 mg) were analyzed under a N_2_ atmosphere.

X-ray photoelectron spectroscopy (XPS) of the char residues was carried out with a VG Escalab Mark II spectrometer (Thermo Scientific, Waltham, MA, USA), using Al Ka excitation radiation (hv = 1253.6 eV).

Raman spectra were obtained using a Stellar-PRO confocal Raman microscopy system (Modu-Laser, LLC, Centerville, UT, USA) with a laser wavelength of 488 nm.

The rheological behavior of PVA-based hydrogels in an oscillating shear field was studied using an Anton Paar shear rheometer (MCR 300) equipped with parallel plate geometry (diameter = 25 mm). Frequency sweep experiments in the range of 0.01 to 100 rad s^−1^ were performed under an air atmosphere, at ambient temperature, and with a strain amplitude of 0.1%.

The mechanical properties of all samples were tested on a universal tensile testing machine, according to the ASTM D-882, with a tensile speed of 20 mm/min.

Water retention: Samples 1~6 were prepared according to Table 1. The water retention capacity of the samples was determined by measuring the total water loss with time, whereby the total water loss was calculated from the initial weight and the final weight (at the time where there was no weight change); the time of total water loss was the time period it took to reach a steady weight.

Swellability: A small rectangular piece of sample was then cut from 1 to 6 cm, weighed and submerged in water, and weighed at certain intervals.

The vertical burning test (UL-94) was performed on a CZF-2 instrument (Bobai County, Guangxi, China), according to GB/T 8333-2008, and the dimensions of all samples were 125 mm × 10 mm × 10 mm.

The cone calorimetry test (CCT) was performed on a cone calorimeter (FTT007, Fire Testing Technology Ltd., West Sussex, UK) according to the ISO5660 standard. The specimens (100 mm × 100 mm × 2 mm) were wrapped in aluminum foil and exposed to a heat flux of 35 kW/m^2^.

## 3. Results and Discussion

### 3.1. Characterization of DOPO-VTES

#### FTIR Analysis

**Figure 1:**
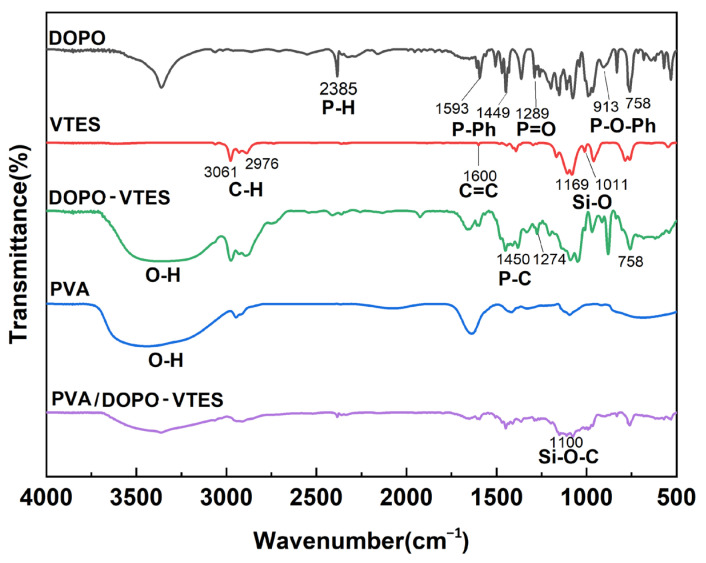
FTIR spectra of DOPO, VTES, DOPO-VTES, PVA and PVA/DOPO-VTES.

The FTIR spectra of DOPO, VTES and DOPO-VTES are shown in Figure 1. The characteristic absorption peak of the P–H bond on DOPO is located at 2385 cm^−1^, the peaks at 1600 cm^−1^, 3061 cm^−1^ and 2976 cm^−1^ belong to C=C and C–H bonds, respectively, and the peaks at 1169 cm^−1^ and 1011 cm^−1^ are assigned to the Si–O bond on VTES. After the addition reaction between the P–H bond on DOPO and unsaturated C=C double bonds on VTES, the P–H bond disappeared and a new absorption peak of the P–C bond appeared at 1450 cm^−1^. This indicates that DOPO underwent an addition reaction with VTES [18]. To further characterize this addition reaction between DOPO and VTES, nuclear magnetic resonance (^1^H NMR and ^29^Si NMR) spectra were used for the characterization of the reaction products.

**Figure 2:**
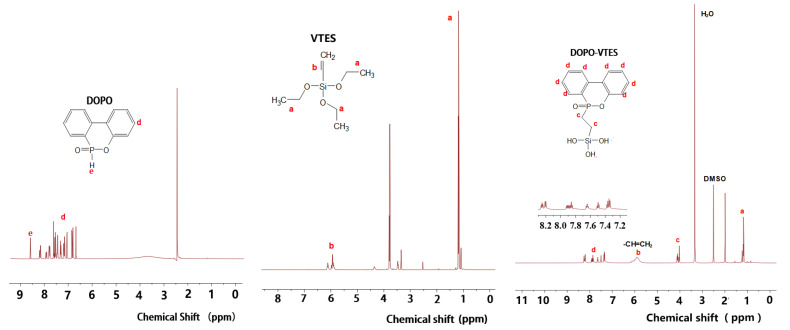
^1^H NMR spectra of DOPO, VTES and DOPO-VTES.

**Figure 3:**
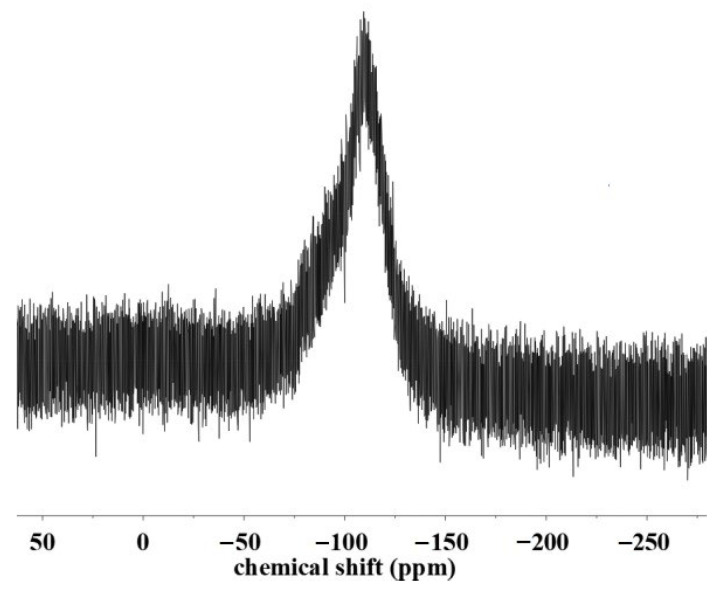
^29^Si-NMR spectra of DOPO-VTES.

Figure 2 shows the proton nuclear magnetic resonance (^1^H NMR) spectra of DOPO, VTES and DOPO-VTES. Compared with DOPO and VTES, for DOPO-VTES, the signal of P–H at 8.68 ppm (peak e) for DOPO almost completely disappeared, and a very small amount of the unreacted vinyl group at 5.9–6.2 ppm (peaks b) for VTES was observed. The peak located at 7.3–8.31 ppm (peaks d) was generated by the ^1^H proton on the biphenyl ring, while the peak at 4.0–4.3 ppm (peaks c) was generated by the ^1^H proton on the methylene group attached to P. The ^1^H NMR characteristics of DOPO-VTES are consistent with Zhang’s work [19]. The ^29^Si-NMR spectra of DOPO-VTES are shown in Figure 3. The signals at −100 ppm were assigned to the Si–OH in DOPO-VTES, and the chemical shifts at −100 ppm represent the T unit, which belong to the –Si(OH)_3_ structure.

### 3.2. Cross-Linking of PVA with DOPO-VTES

**Scheme 2:**
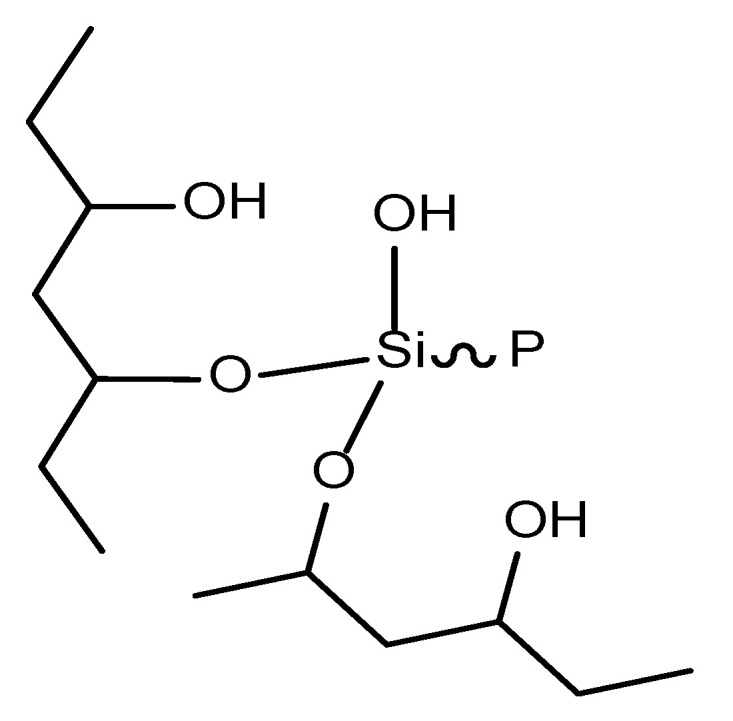
The cross-linked structure of PVA and DOPO-VTES.

The molecular structure of PVA was rich in hydroxyl groups. The hydrolysis of DOPO-VTES produced silicon hydroxyl groups, which were dehydrated and cross-linked with the hydroxyl groups of PVA to introduce P–Si flame retardant into the PVA molecular chain [20]. The infrared spectra of DOPO, DOPO-VTES and PVA/DO-VTES are presented in Figure 1. It was found that there was a large and wide absorption peak for DOPO-VTES and PVA around 3400 cm^−1^ separately, which is the characteristic absorption peak of the hydroxyl group, indicating that DOPO-VTES and PVA contain hydroxyl groups. However, the peak intensity of the hydroxyl group in the spectrum of PVA/DOPO-VTES was weakened. Additionally, there is a wide absorption peak around 1100 cm^−1^, belonging to the Si–O–C peak, which indicated that PVA/DOPO-VTES contains an Si–O–C bond. These characteristics proved that Si–OH in DOPO-VTES and OH in PVA probably reacted and crossed. Their structure is schematically shown as Scheme 2.

#### 3.2.1. Rheological Properties

**Figure 4:**
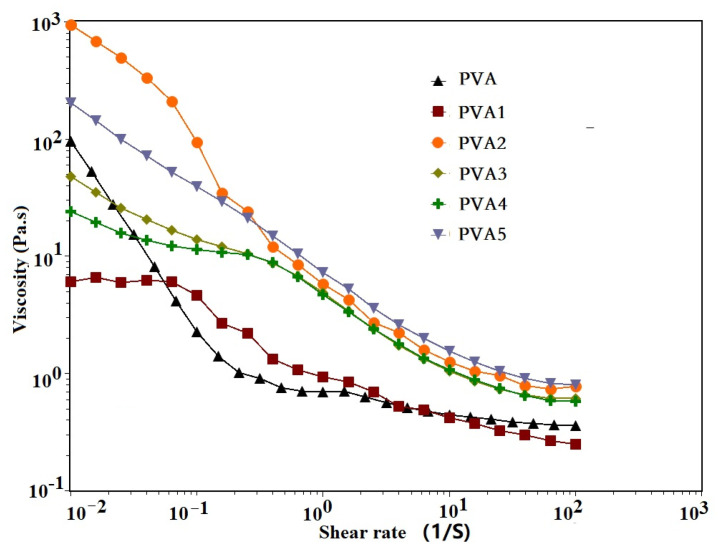
The viscosity curves of the PVA and PVA composite films with the shear rate.

PVA coating was applied onto some substances to prevent them from catching fire, and this coating application required low viscosity. Factors affecting the rheological properties of PVA were discussed, such as the temperature, the shear rate, a cross-linking agent and an additive. Here, the influence of a cross-linking agent and an additive on the viscosity of PVA will be discussed. It could be observed from Figure 4 that the viscosity of all samples showed a decreasing trend when the shear rate increased, which showed the characteristics of pseudoplastic fluid. At shear rates ranging from1 10^−1^ 1/S to 10^0^ 1/S, PVA1 was more viscous than pure PVA, due to the C–OH in PVA cross-linking with the Si–OH in the flame retardant DOPO-VTES, which increased the force between the PVA molecules. For PVA2 to PVA5 with α-ZrP, an increase in viscosity was observed compared with pure PVA and PVA1, because of α-ZrP’s lamellar structure. The friction force between the PVA molecules was increased under certain shear action. The cross-linking agent and additive improved the viscosity of the PVA, but did not affect brush application.

#### 3.2.2. DSC

The crystallinity (Xc) of the PVA composite was calculated as the following [21]:

Xc = (∆Hc/∆Ho) × 100%

∆Hc represents the melt enthalpy of the PVA composites; ∆Ho represents the melt enthalpy of 100% PVA, which is 168 J/g [22].The crystallinity calculated by the formula is shown in Table 2.

**Figure 5:**
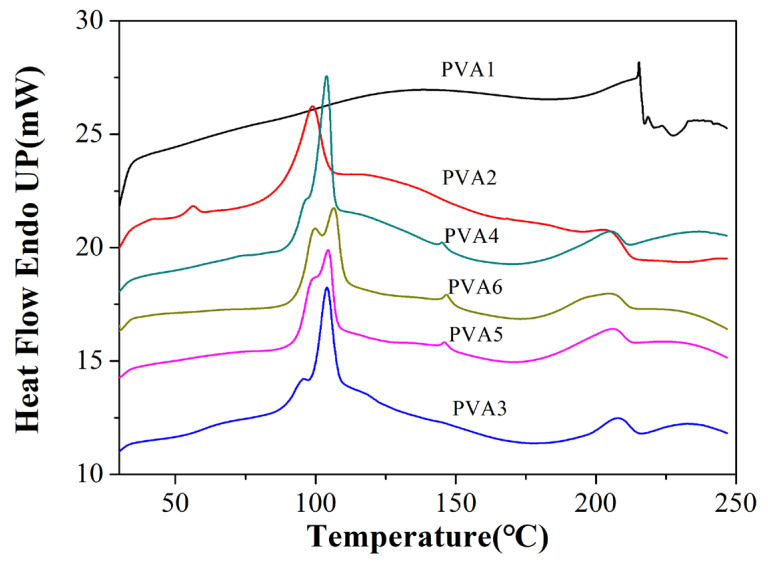
DSC curves of the capacity of the PVA and PVA composite films.

According to Figure 5, the crystallization temperature of PVA was approximately 210–220 °C [23]. After adding the DOPO-VTES to PVA1, the crystallization temperature was around 185–215 °C, and the crystallinity was reduced from 16% to 14.8%. This reduction in crystallinity could be attributed to the cross-linking between PVA and DOPO-VTES, which restricted the activity of PVA molecules and decreased the crystallization ability. The addition of α-ZrP increased the crystallinity from 14.8% to 42% for PVA1, PVA2 and PVA3. α-ZrP acted as a crystalline nucleating agent [24] in PVA and improved the crystallinity of the PVA composite films. However, in Table 2, the crystallinity was greatly reduced for PVA4 and PVA5, which was due to α-ZrP inhibiting the movement of PVA molecules. Figure 5 shows a sharp melting point at 90 °C, which probably occurred owing to DOPO-VTES’s decomposed peak on the PVA chain.

### 3.3. Basic Properties of the PVA Composite Films

#### 3.3.1. Water Retention Capacity and Water Absorption Capacity

**Figure 6:**
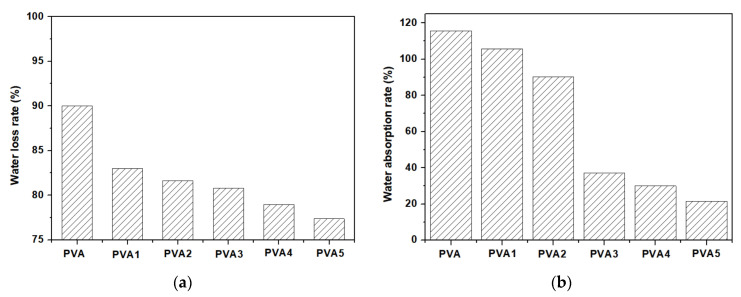
Water retention capacity curve (**a**) and water absorption capacity curve (**b**) of the PVA films.

The water loss rate was used to analyze the water retention capacity of the PVA films, where the total water loss was calculated from the initial weight and the final weight (at the time where there was no weight change); the time of total water loss was the time period required to reach a steady weight. The water retention curves of the PVA films are shown in Figure 6a, which shows the water loss from the films to the environment. Figure 6b illustrates the water absorption of the PVA films from the environment. The PVA molecule contained a large number of hydroxyl groups (–OH), which were hydrophilic and had a certain water retention capacity. After cross-linking with DOPO-VTES, the number of –OH on the PVA molecule decreased, forming a cross-linking reticulation, and the water loss capacity decreased, so PVA > PVA1. The water loss capability of the PVA films containing α-ZrP was reduced by the barrier effect of the α-ZrP flakes. For the same reason, water absorption was the opposite of water loss.

**Figure 7:**
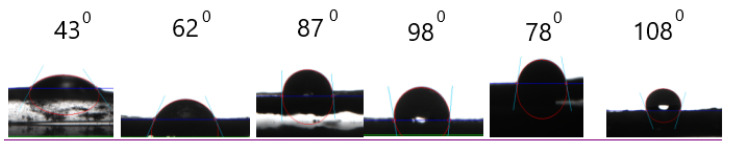
Water contact angles of the PVA films.

Due to the consumption of –OH groups resulting in hydrophobicity, contact angle measurements were used to analyze changes in hydrophilicity. It could be seen from Figure 7 that the contact angle increased with increasing α-ZrP content, which was attributed to the depletion of –OH groups by PVA cross-linked with DOPO-VTES, and the improvement in the surface roughness of the PVA film by α-ZrP. This indicates that α-ZrP could improve the water retention capacity of PVA.

#### 3.3.2. Mechanical Properties

**Figure 8:**
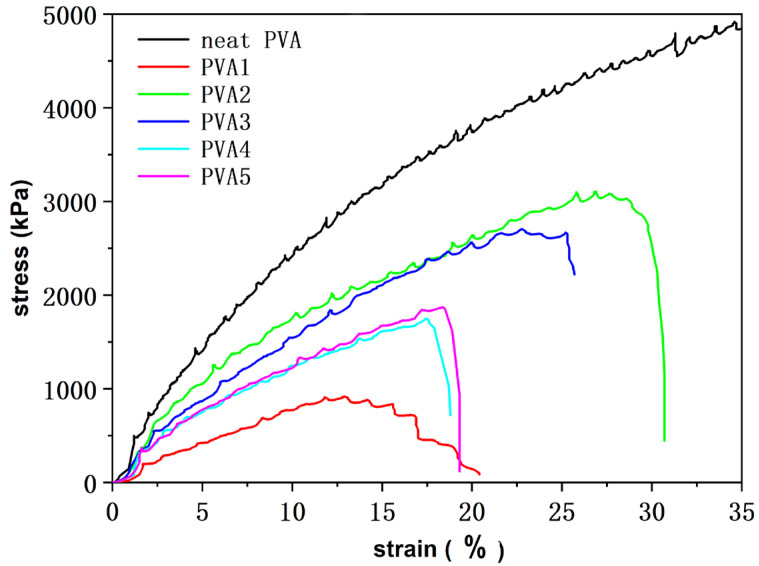
Tensile stress–strain curves of the PVA composite films.

The tensile stress–strain curves of the PVA composite films are shown in Figure 8. Pure PVA had good toughness, and the toughness of the PVA decreased when PVA cross-linked with the flame retardant DOPO-VTES, because PVA cross-linked with DOPO-VTES formed a cross-link network structure. When stretched, the network structure was vulnerable to damage, and the tensile strength decreased compared to PVA. In addition, the toughness of the composite films with added α-ZrP was better than PVA1. Among them, PVA2 exhibited the highest tensile strength, reaching 3000 kPa. However, for PVA3, PVA4 and PVA5, α-ZrP hindered the cross-linking of PVA macromolecular chains with DOPO-VTES, so that more voids and phase separation were formed between the PVA and filler, making the material loose and the mechanical properties decrease. Therefore, for PVA2, the cross-linking of PVA with DOPO-VTES formed a more stable cross-linked structure and maintained good mechanical properties.

#### 3.3.3. Thermal Stability

**Figure 9:**
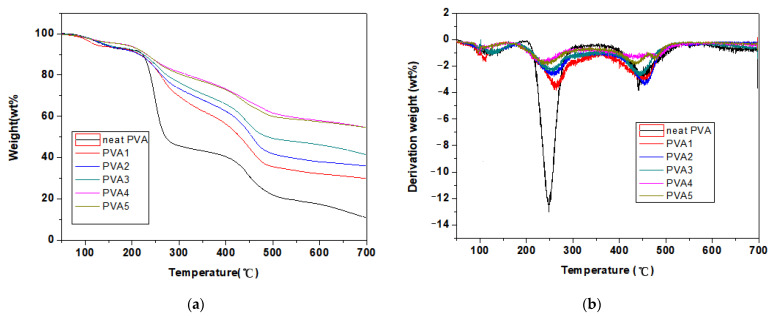
TGA (**a**) and DTG (**b**) curves of the PVA and PVA composite films in N_2_.

The TG and DTG curves of the PVA and PVA composite films under a nitrogen atmosphere are shown in Figure 9, and the relevant data are shown in Table 3. Two thermal degradation rate peaks appeared in the TGA of the PVA. The first stage, at 220–270 °C, was caused by the elimination of the side hydroxyl groups, with a loss of about 50% of the mass, and produced mainly pyrolysis products, such as water, isolated and conjugated polyenes, aldehydes, and ketones; the second stage, at 400–500 °C, was dominated by random chain cracking and cyclization reactions [25]. The initial decomposition temperature (T_5%_) of the pure PVA was 125.5 °C, whereas the residual carbon at 700 °C was only 11%. For PVA1, the degradation temperature was close to pure PVA, but the residual carbon reached 30%, which was due to DOPO-VTES cross-linking with PVA, improving the residual char. After the addition of α-ZrP to PVA/DOPO-VTES, the initial decomposition temperature of PVA increased from 125 °C to 150 °C, and the residual char increased from 30% to 54%. α-ZrP improved the thermal stability and the residual char of PVA/DOPO-VTES.

The decomposition rates of PVA were in the order of PVA > PVA/DOPO-VTES > PVA/DOPO-VTES/α-ZrP. This indicates that DOPO-VTES inhibited the decomposition of PVA, but the decomposition rate of PVA2 was higher than that of PVA1, which was attributed to the catalytic effect of α-ZrP in promoting the decomposition of PVA and the formation of char during the combustion process.

### 3.4. Flammability

#### 3.4.1. Vertical Burning Test (UL-94)

The flammability of the PVA composite films was evaluated by the vertical burning UL-94 test. It can be seen from Table 4 that pure PVA is flammable and burns violently, and it cannot pass the UL-94 level test, while PVA1 with DOPO-VTES produced melt dripping, ignited cotton during combustion and reached a UL-94 V-1 rating. PVA2, PVA3, PVA4, PVA5 with DOPO-VTES and α-ZrP did not produce melt dripping during combustion and reached a UL-94 V-0 rating. Compared with the above samples, DOPO-VTES has a certain flame-retardant effect on PVA. The addition of α-ZrP further improved the flame-retardant effect of DOPO-VTES on PVA, and so the combination of DOPO-VTES with α-ZrP exhibits a synergistic flame-retardant effect on PVA.

#### 3.4.2. Cone Calorimetry Test (CCT)

**Figure 10:**
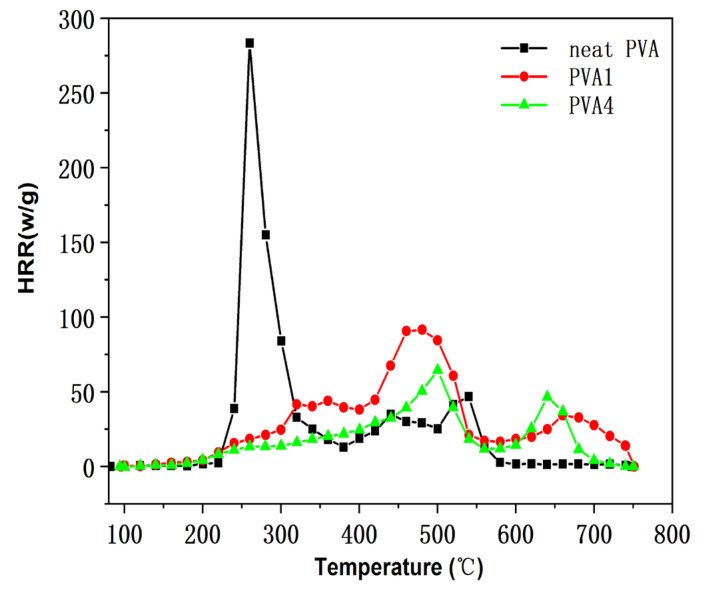
Heat release rate of the PVA and PVA composite films.

The CCT is a method of rapidly obtaining the combustion properties of materials from a small number of samples, providing key data such as the heat release rate (HRR) and total heat release (THR). Figure 10 shows the HRR curves of pure PVA and flame-retardant PVA composite films, and the main information of the combustion data obtained from the HRR curves is shown in Table 5. Generally, the higher the HRR value was, the more heat was fed back to the material surface during combustion, accelerating the flame spread [26]. The HRR of PVA was 312.60 kw/m^2^ and the THR was 18.7 MJ/m^2^. The introduction of DOPO-VTES into PVA reduced the release rate and the total heat release of the PVA; however, PVA1 had an HRR of 92.9 kw/m^2^ and a THR of 19.0 MJ/m^2^. By adding α-ZrP to PVA1, the HRR and THR of the PVA4 composites were further reduced to 64.9% and 11.7 KJ/g, respectively. These results are consistent with those of TGA, indicating that DOPO-VTES had a certain flame-retardant effect on PVA, and the combination DOPO-VTES and α-ZrP showed a cooperative effect on PVA.

### 3.5. Flame Retardant Mechanisms

#### 3.5.1. EDS Analysis of the Char Layer

**Figure 11:**
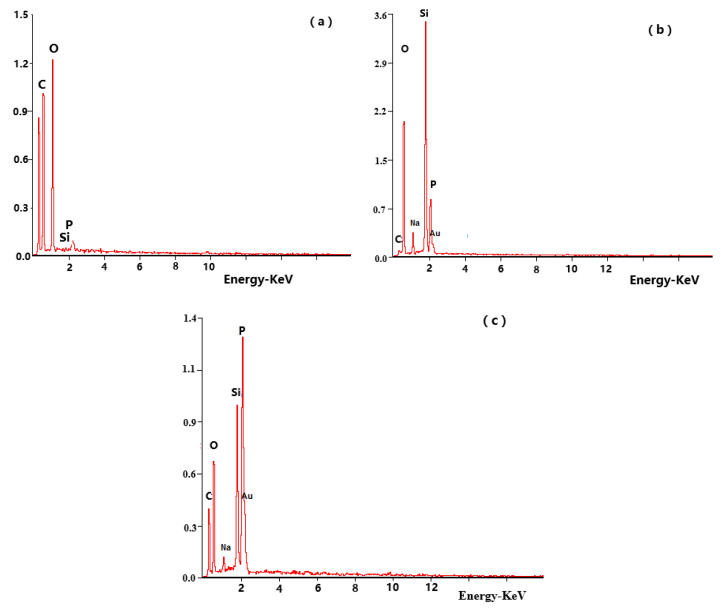
EDS spectra of (**a**) the neat PVA, (**b**) PVA1 and (**c**) PVA4.

EDS analysis was used to determine the elemental composition of the residual char layer of the PVA composite films after the UL-94 test. It could be seen from Figure 11 that the residual char layer contained C, O, Si, P and other elements. The corresponding content is listed in Table 6. The PVA and the PVA composite films produced carbon and carbon compounds containing oxygen during combustion. The combination of DOPO-VTES and α-ZrP in PVA resulted in an increase in phosphorus content and a decrease in oxygen content due to DOPO in the DOPO-VTES exhibiting a mainly gas-phase flame retardant, which yielded a PO radical; VTES in the DOPO-VTES producing silicon dioxide (SiO_2_), which acted as a thermal insulator; and the α-ZrP catalyzing the carbonization of the PVA. At the same time, from a theoretical perspective, actual phosphorous-containing content was decreased for PVA1 and PVA4 because the pyrolysis of DOPO produced phosphorous-containing free radicals (PO·, PO_2_·and HPO_2_·) to capture H· and OH· radicals in the flame zone to suppress a combustion reaction [27]. Therefore, DOPO-VTES exhibited a gas-phase flame-retardant mechanism.

#### 3.5.2. XPS

**Figure 12:**
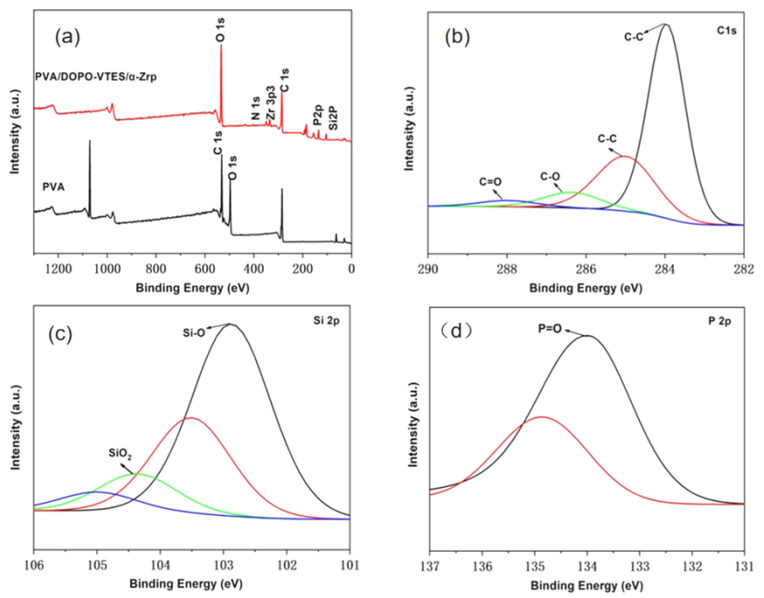
XPS spectra of residue chars for the flame-retardant PVA composite films; (**a**) Binding energy of the flame-retardant PVA composite films; (**b**) Binding energy of C_1s_; (**c**) Binding energy of Si_2P_; (**d**) Binding energy of P_2P_.

XPS was used to analyze the elemental composition of the char residues and the forming bonding mode. Combining the data in Figure 12 and Table 7, it could be seen from Figure 12a that the binding energies of the flame-retardant PVA composite films peaked at 533.1 eV, 286.4 eV, 134.1 eV and 104.3 eV, respectively, which could be assigned to O_1_s, C_1_s, P_2P_ and Si_2P_. The relative contents of the C, P, O and Si elements were 3.08%, 8.80%, 71.97% and 16.15%, respectively. The elemental compositions indicated that the flame-retardant PVA composite films contained elements of the DOPO-VTES and α-ZrP. For the C_1_s spectrum in Figure 12b, the peaks at 285.0, 286.4 and 288.0 eV corresponded to C–C, C–O and C=O chemical bonds, respectively, indicating the flame-retardant PVA composite films produced charcoal and carbon oxides while burning, of which CO_2_, as a non-flammable gas, played a role in diluting the oxygen concentration in the air. For the Si_2_p spectrum in Figure 12c, the peaks at 102.9 and 104.4 eV corresponded to the Si–O chemical bond and SiO_2_, respectively. SiO_2_ on the surface of the char layer behaved as a heat insulator to hinder heat transformation [28]. For the P_2P_ spectrum in Figure 12d, the peak at 134.1 eV corresponded to the P=O chemical bond, and compounds containing phosphorus helped to catalyze the char formation of PVA. The XPS data exhibited that DOPO-VTES acted through a condensed-phase flame-retardant mechanism.

#### 3.5.3. SEM of the Residual Char

**Figure 13:**
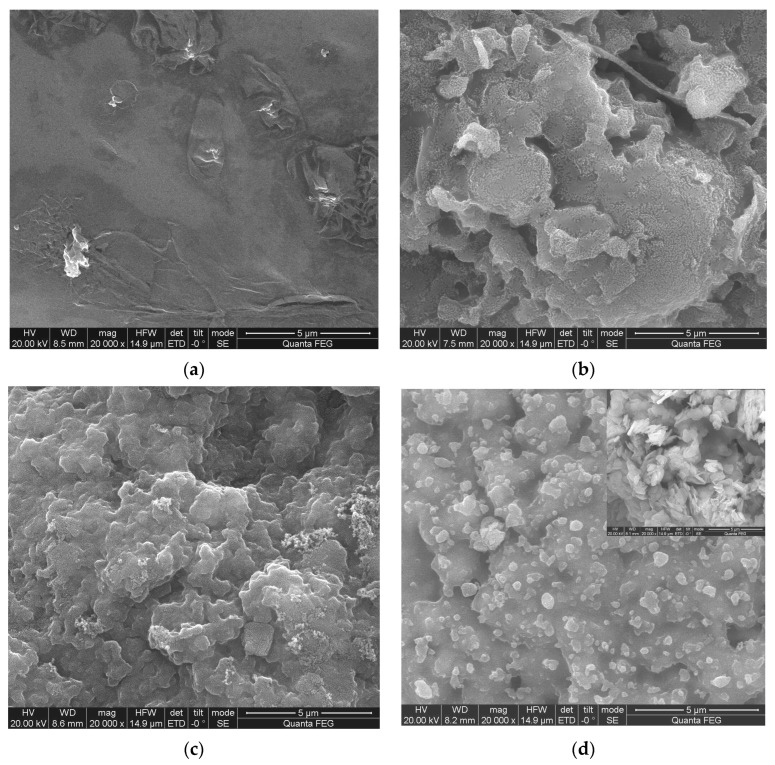
SEM images of the residual char for (**a**) PVA, (**b**) PVA1, (**c**) PVA 4 and (**d**) PVA 5.

Figure 13 shows the morphologies of the char layer of the PVA composite films after UL-94 testing, from which it could be seen that pure PVA could not pass the UL-94 test and could not form an effective char layer, while PVA1, PVA 4 and PVA 5 with DOPO-VTES could form a char layer, with α-ZrP covering the surface of the carbon layer. The combination of DOPO-VTES and α-ZrP formed a continuous and dense char layer. The possible flame-retardant mode of action at play was that DOPO in the DOPO-VTES acted as a mainly gas-phase flame retardant, which yielded a PO radical; VTES in the DOPO-VTES produced silicon dioxide (SiO_2_), which acted as a thermal insulator; and α-ZrP catalyzed the carbonization of the PVA. α-ZrP is a type of two-dimensional layered nanomaterial. There are a lot of Brønsted and Lewis acid points in the interlayer of α-ZrP, which can catalyze PVA cross-linking to form carbon during high-temperature combustion and form a “barrier” to block the transport of combustible gas, oxygen and heat. The combination of DOPO-VTES and α-ZrP had a cooperative flame-retardant effect on PVA [29,30].

#### 3.5.4. Raman Spectroscopy of the Residual Char

**Figure 14:**
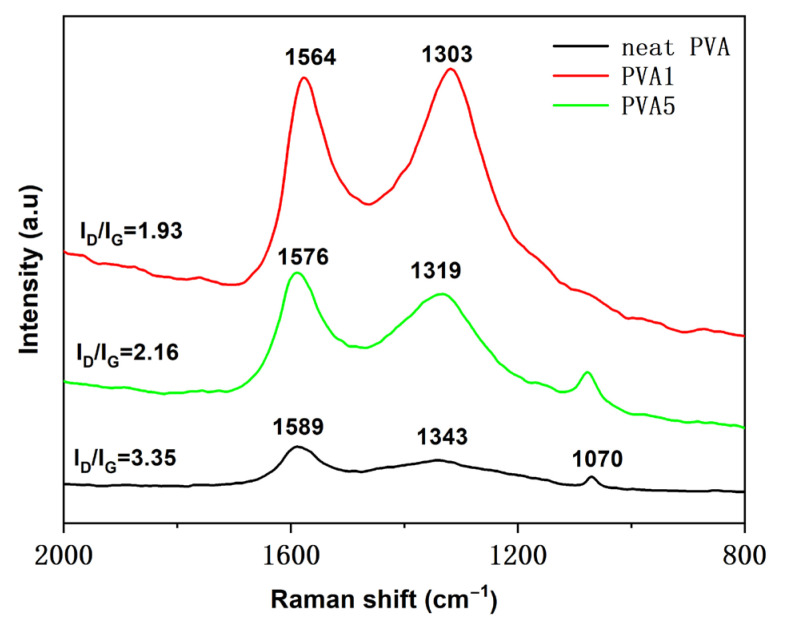
Raman spectroscopy of the residual char for the PVA composite films.

To investigate the degree of graphitization, the pure PVA, PVA1 and PVA5 samples after conical calorimetry were tested using Raman spectroscopy, where the D peak (1360 cm^−1^) was generated by disordered graphite, and the G peak (1580 cm^−1^) was generated by graphite. The D and G peak area ratio was used to measure the graphitization degree of residual char, which meant the smaller the I_D_/I_G_, the higher the graphitization degree [31]. As shown in Figure 14 for PVA, PVA1, PVA5 and I_D_/I_G_, the ratios were 3.35, 2.16 and 1.93, respectively. After comparing the I_D_/I_G_ values of the three samples, it was obvious that the graphitization degree gradually increased. The increasing trend of the degree of graphitized residual charcoal was consistent with the increasing trend of the amount of residual charcoal above the TGA of the samples, forming a continuous and dense charcoal layer, which effectively isolated oxygen and heat exchanges and thus improved the flame retardancy of the PVA composite films. This indicated that the addition of DOPO-VTES and α-ZrP promoted the formation of a continuous and dense char layer during the combustion for PVA.

## 4. Conclusions

The phosphorus-silica flame retardant DOPO-VTES was synthesized through the addition reaction of the P–H bond on DOPO and unsaturated carbon–carbon double bonds on VTES. Then, the DOPO-VTES and α-ZrP were introduced to PVA to form a PVA flame-retardant film. The CCT results showed that the HRR and THR of the flame-retardant PVA composite films were significantly lower than those of pure PVA, and the TGA results indicated that the residual char of the flame-retardant PVA composites was significantly higher than that of pure PVA, which indicated that the combination of DOPO-VTES and α-ZrP had a cooperative flame-retardant effect on PVA. The cooperative flame-retardant mode of action of DOPO-VTES and α-ZrP may be due to DOPO in the DOPO-VTES, which acted as a mainly gas-phase flame retardant yielding a PO radical; VTES in the DOPO-VTES producing silicon dioxide (SiO_2_), which acted as a thermal insulator; and α-ZrP catalyzing the carbonization of the PVA. The formed char layer by the combination of DOPO-VTES and α-ZrP effectively inhibited oxygen and heat exchange, thus achieving flame retardancy. This flame-retardant film for PVA is expected to find a wide range of applications, especially in food packaging materials, electrical and electronic appliances, and lithium-ion battery separators.

## Figures and Tables

**Table 1:** Formulations of the flame-retardant PVA composite films.

Component			
Sample	PVA Solution (8 wt%/g)	DOPO-VTES (g)	α-ZrP (g)
Neat PVA	100	0	0
PVA1	100	50	0
PVA2	100	50	2.5
PVA3	100	50	5
PVA4	100	50	10
PVA5	100	50	15

**Table 2:** Crystallization performance parameters of the PVA composites.

Sample	Temperature (°C)	∆Hc/Jg^−1^	Xc/%
PVA	222.5	27.7	16.0
PVA1	206.2	25.0	14.8
PVA2	209.8	62.7	37.3
PVA3	210.4	70.0	42.0
PVA4	209.9	41.0	24.4
PVA5	206.4	7.5	4.4

**Table 3:** TGA data of the PVA and PVA composite films in N_2_.

Sample	T_5%_ (°C)	R_max_ (%·min^−1^)	T_Max_ (°C)	Residue at 700 °C (%)
PVA	125.5	11.9	250.2	11.0
PVA1	125.8	2.9	250.1	30.0
PVA2	125.6	3.3	450.1	30.6
PVA3	149.9	2.7	450.1	41.5
PVA4	150.1	1.7	450.1	54.6
PVA5	150.0	1.8	450.1	54.7

**Table 4:** Test results of the PVA composite film.

Sample	UL-94 Test (3.2 mm)				
	t_1_ [s]	t_2_ [s]	Dripping	Ignites Cotton	Rating
neat PVA	30	15	Yes	Yes	NR
PVA1	8	14	No	Yes	V-1
PVA2	5	9	No	No	V-0
PVA3	5	6	No	No	V-0
PVA4	6	8	No	No	V-0
PVA5	7	4	No	No	V-0

**Table 5:** CCT data of the PVA composite films.

Sample	HRR (kw/m^2^)	THR (MJ/m^2^)	Temperature (°C)
Neat PVA	312.6	18.7	262.7
PVA1	92.9	19.0	463.9
PVA4	64.9	11.7	496.8

**Table 6:** EDS data of the PVA composite films.

Sample	C (wt%)	O (wt%)	Si (wt%)	P (wt%)	Real P (At%)	Theoretical P (At%)
Neat PVA	45.02	54.75	0	0	0	0
PVA1	7.95	50.40	27.02	10.49	6.37	8.0
PVA4	52.48	23.71	7.25	15.46	7.50	9.0

**Table 7:** Element relative contents of the char layer for the flame-retardant PVA composite films.

Element	C	P	O	Si
Peak BE (ev)	283.96	134.08	531.89	102.87
Atomic percentage (%)	3.08	8.80	71.97	16.15

## Data Availability

The original contributions presented in the study are included in the article material, further inquiries can be directed to the corresponding author.
